# The Imperial College Storm Model (IRIS) Dataset

**DOI:** 10.1038/s41597-024-03250-y

**Published:** 2024-04-24

**Authors:** Nathan Sparks, Ralf Toumi

**Affiliations:** Imperial College London, Department of Physics, London, SW7 2AZ UK

**Keywords:** Natural hazards, Climate sciences

## Abstract

Assessing tropical cyclone risk on a global scale given the infrequency of landfalling tropical cyclones (TC) and the short period of reliable observations remains a challenge. Synthetic tropical cyclone datasets can help overcome these problems. Here we present a new global dataset created by IRIS, the ImpeRIal college Storm model. IRIS is novel because, unlike other synthetic TC models, it only simulates the decay from the point of lifetime maximum intensity. This minimises the bias in the dataset. It takes input from 42 years of observed tropical cyclones and creates a 10,000 year synthetic dataset of wind speed which is then validated against the observations. IRIS captures important statistical characteristics of the observed data. The return periods of the landfall maximum wind speed are realistic globally.

## Background & Summary

Tropical cyclones (TCs) are extreme weather systems which, when they make landfall, can become a deadly natural disaster causing deaths and billions of dollars worth of loss per year^1^. Under climate change, TCs, also known as hurricanes and typhoons, are expected to become more intense and therefore even more damaging^2,3^. Reliable estimates of TC risk are desirable and can help mitigate TC hazard impact. However, TCs are relatively infrequent events, and those which make landfall are even more scarce. This makes assessing even the current climate baseline risk a challenge. The relatively short length of the reliable historical record compounds this difficulty.

A popular approach to this problem is creating synthetic sets of TCs which share important properties of observed TCs. These datasets may be arbitrarily long and therefore overcome the problem of scarcity in the observations. Generating large sets of synthetic TCs using full-physics numerical simulations is problematic for two main reasons. Firstly, running global climate simulations at resolutions which resolve TCs effectively is very computationally costly hence it is not feasible to generate the thousands of years required by the risk modelling community using this method. Secondly, there are still many biases present in TCs simulated in climate models.

Statistical methods are therefore often preferred and have been popular in recent decades. An early model for the North Atlantic^4^ re-sampled observed genesis locations and derived stochastic tracks from these based on models fitted per 5 degree box. Hybrid statistical-physical models couple statistical elements (e.g. track) to physics simulations^5^ (e.g. intensity). These models may require a large quantity of input data and be computationally expensive. Furthermore, landfall statistics may not be well-represented and are subjected to bias correction^6^. A physics-based statistical downscaling model forced by environmental fields reasonably reproduces observed landfall return curves in some global sub basins^7^. Fully statistical models have also been developed at the regional scale^8,9^ which are quick and cheap to run. A global model has also been recently proposed^10^. All the above models simulate TCs beginning at the point of genesis, when the TC first comes into being as a weak storm. Errors or biases in the simulation of genesis, intensification, track, and decay of TCs are all likely to lead to biases in the simulated landfall statistics.

The main purpose of these types of models is to simulate TCs at landfall. A key insight^11^ is that landfall maximum wind speed can be reliably estimated from the position and value of the life-time maximum intensity. The problem then becomes a decay only problem. This assumption short circuits much of the life-cycle which avoids the accumulation of errors occurring in other models. It also allows us to simulate other important variables such as the size and pressure-wind relationship specifically for this critical decay phase of the life-cycle, rather than assuming one relationship throughout when very different statistical relations may apply in different phases of the TC life-cycle. This is a novel approach not followed by other models^4,6,8–10^. Here we present a new model, the ImpeRIal college Storm model (IRIS), to test and demonstrate the utility of this assumption.

## Methods

### Model overview

IRIS is a stochastic TC wind model capable of producing thousands of years’ worth of TCs at relatively low computational cost (the dataset described here was generated in a few hours on a 40-core workstation). A key novel component of IRIS is that only post-LMI TC tracks are simulated. This mean no biases or errors associated with the simulation of the intensification stage of TC development can affect the model. Meanwhile the most hazardous and critical, post-LMI, stage of the TC, including landfall, receives full focus. IRIS simulates basins and years independently. For a given basin and model year, IRIS randomly samples post-LMI tracks from the entire 42-year historical record and perturbs and extends these tracks as required. Each TC track is assigned a new initial maximum wind speed (LMI). From LMI a decay parameterisation governs the maximum wind speed until dissipation. Decay over land and ocean are different. A size is calculated by a parametric radial wind profile for each TC which evolves along its track. Finally, IRIS simulates the minimum pressure. Running IRIS proceeds in three main stages: (1) Data preparation, (2) parameter fitting, and (3) simulation. These stages are described below.

### Data preparation

IRIS requires input in the form of: historical TC track data, climatological surface pressure, climatological wind speeds at the TC steering level, and fields of climatological MPI. We describe how each of these are obtained.

#### TC track data

**Fig. 1:**
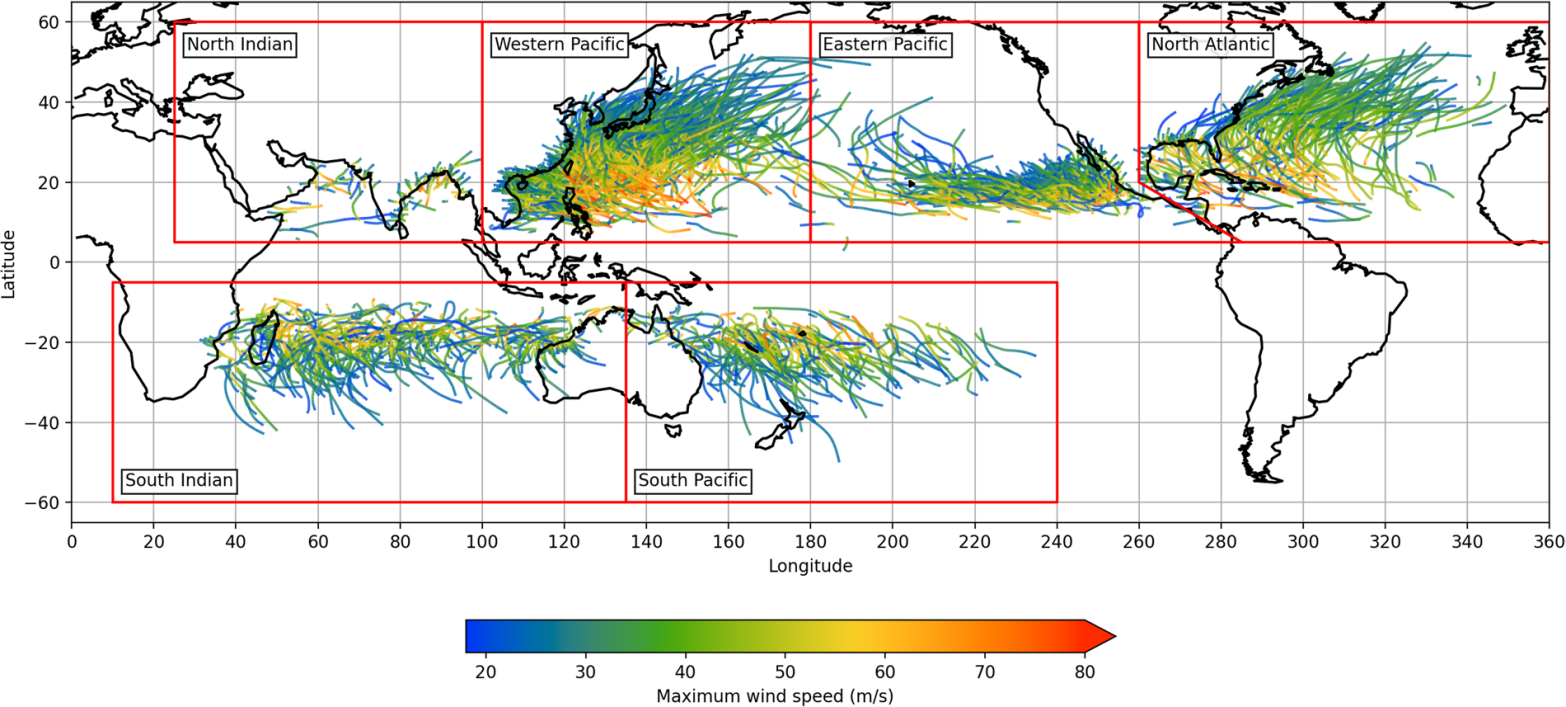
TC track input data from IBTrACS after processing.

We extract TC track data from the International Best Track Archive for Climate Stewardship (IBTrACS, v04r00) World Meteorological Organisation data^12,13^. Tracks were restricted to seasons in the range 1980 to 2021, a period of 42 years, to coincide with the era of satellite observations. We use data from the U.S. agencies, which report wind speeds as 1-minute average sustained winds and span the 6 main TC basins. Irregular time steps are removed such that all data are sampled at 3 hour intervals. Only tropical storm systems which reach at least category 1 wind speeds at LMI are considered in this model, extra- and post-tropical systems are excluded. The LMI of each storm is identified and time steps prior to the last occurrence of the LMI are excluded. In this sense all the tracks in the input data are decaying. Finally, decaying TC tracks are terminated at the point the wind speed falls below the tropical storm threshold. Figure 1 shows the TC track input data after processing.

#### Meteorological fields

The ERA5 reanalysis product provided monthly mean sea level pressure^14^, and wind velocity at 500 hPa^15^. These data were obtained at quarter degree resolution and used to provide climatological monthly mean values calculated over the observation period for the input TCs described above (1980–2021).

#### Potential intensity

The principal physical constraint on the model is through the thermodynamic state as defined by the potential intensity which depends on the sea surface temperature (SST) and the vertical temperature and humidity profile. Daily maps of PI were calculated for the observation period. We used the modified algorithm^16,17^ based on the theoretical model of Emanuel^18^. This algorithm requires SST, surface pressure^19^, and vertical profiles of temperature and humidity^20^. ERA5 data at half degree resolution was used for all these variables. After calculating daily values, monthly mean and climatological monthly mean values were calculated. PI values maybe affected by the presence TCs in the reanalysis data, but we expect this effect to be small in the monthly means and climatology.

### Parameter fitting

#### Count

Annual basin count distributions are approximately Poisson with Poisson parameter, *λ*, equal to the annual mean count. We can therefore simulate the annual basin count, *n*, as

1 $$n \sim {\rm{Pois}}(\lambda ),$$

where *λ* is the mean annual basin count in the input data.

#### Track

**Fig. 2:**
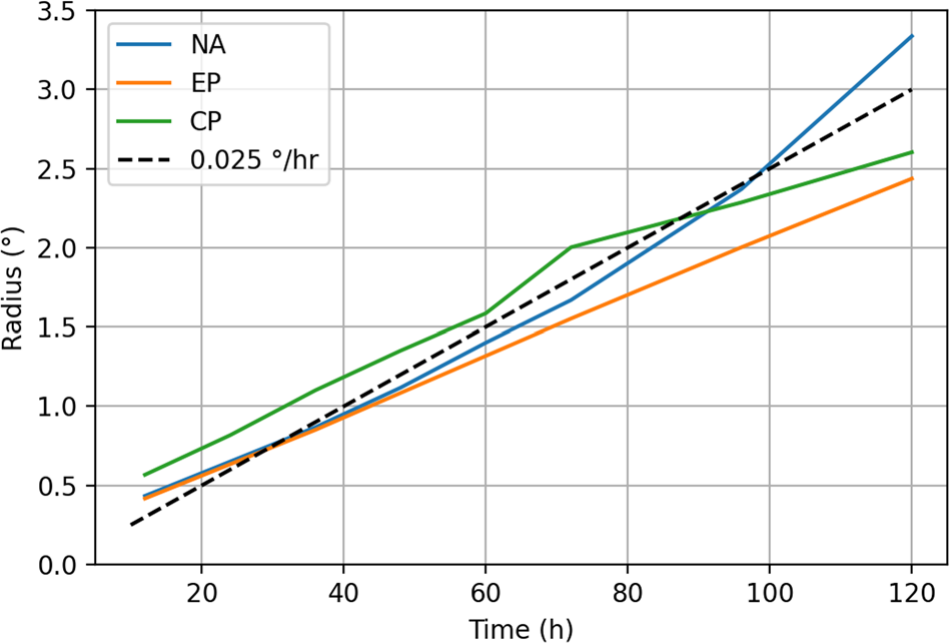
Growth of the National Hurricane Centre forecast track uncertainty cone with time.

In IRIS, observed “parent” tracks are perturbed to create stochastic “child” tracks. The scale of perturbation is derived from National Hurricane Centre (NHC) forecast cones^21^. These cones describe how the uncertainty of TC centre location typically grows with forecast time in numerical weather prediction models. A cone surrounding a track forecast contains the observed track in two thirds of cases. Figure 2 shows estimates of cone size provided by the NHC as a function of forecast time for North Atlantic, Eastern Pacific and Central Pacific TC forecasts. The rate of cone growth in the three regions is very similar, linear in time, and approximately equal to 0.025 deg/hr which is assumed globally in IRIS.

A “child” track perturbed from its “parent” by a displacement growing in time at a rate drawn from a zero-mean normal distribution with a standard deviation of 0.025 deg/hr (i.e. a constant translation velocity perturbation) will fall within its parent’s cone approximately two thirds of the time. Hence we choose this as the track perturbation model.

The “child” tracks we generate can be considered counterfactual of the historic, “parent”, track.

#### LMI

**Fig. 3:**
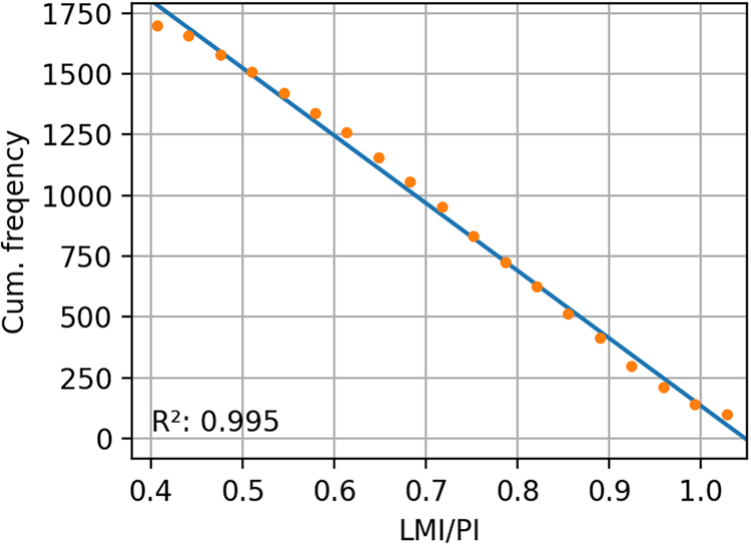
Cumulative frequency of global relative intensity where cumulative frequency is number of TCs exceeding the relative intensity value on the x-axis. R^2^ for least squares linear fit to data over displayed range shown.

For each TC we calculate the PI at the time and location of its LMI. We tested different averaging of PI - monthly mean, monthly mean climatology, monthly maximum, monthly maximum climatology, daily, three days prior to LMI - and found very similar results for all. We confirm that the relative intensity, the ratio of LMI to PI, is uniformly distributed across observations using monthly mean PI values^22^. The cumulative frequency distribution of relative intensity (LMI/PI) is shown in Fig. 3. The cumulative frequency plots and linear fits (R^2^ > 0.99) show that the distribution is indeed highly uniform. The location and value of the relative intensity is therefore the key physical basis in the model. This has the additional benefit that the observational data from the satellite era are most robust at LMI and the application of PI (relative intensity) is most appropriate at the point of LMI in the TC life-cycle.

We can then define the relative intensity as uniformly distributed:

2 $$\frac{LMI}{PI} \sim {\mathscr{U}}(0.4,1.0)$$

where the upper bound is chosen as 1.0, the theoretical maximum, and the lower bound chosen as 0.4 which is less than 99% of the observed LMI/PI.

#### Decay

We calculate the TC decay from LMI assuming a physically informed algebraic decay, recently shown to be suitable over ocean^11^ and land^23^. Maximum surface wind speed as a function of time, *Vmax*(*t*), is given by,

3 $$Vmax(t)={\left[1/LMI+\kappa t\right]}^{-1},$$

where LMI is the speed at t = 0 and *k* is a decay coefficient treated as a constant for a given TC decay. In previous work Wang and Toumi^11^ have shown no simple dependence of *k* on environmental conditions such as the wind shear or SST. It can thus be simulated as a stochastic process. The key determinants for the landfall wind speed are the LMI and decay time. The decay time itself is closely controlled by the location of LMI. *k* is much larger over land and treated separately.

First we separate each TC track into “legs” which occur exclusively over either ocean or over land. We then fit Eq. 3 to each decay leg using a least squares method to estimate *k*. For ocean decay we find that log *k* is distributed approximately normally with a dependence on LMI:

4 $$log\,\kappa ={m}_{a}LMI+{c}_{a}+{\varepsilon }_{a},{\varepsilon }_{a} \sim {\mathscr{N}}(0,{\sigma }_{a}^{2}).$$

**Fig. 4:**
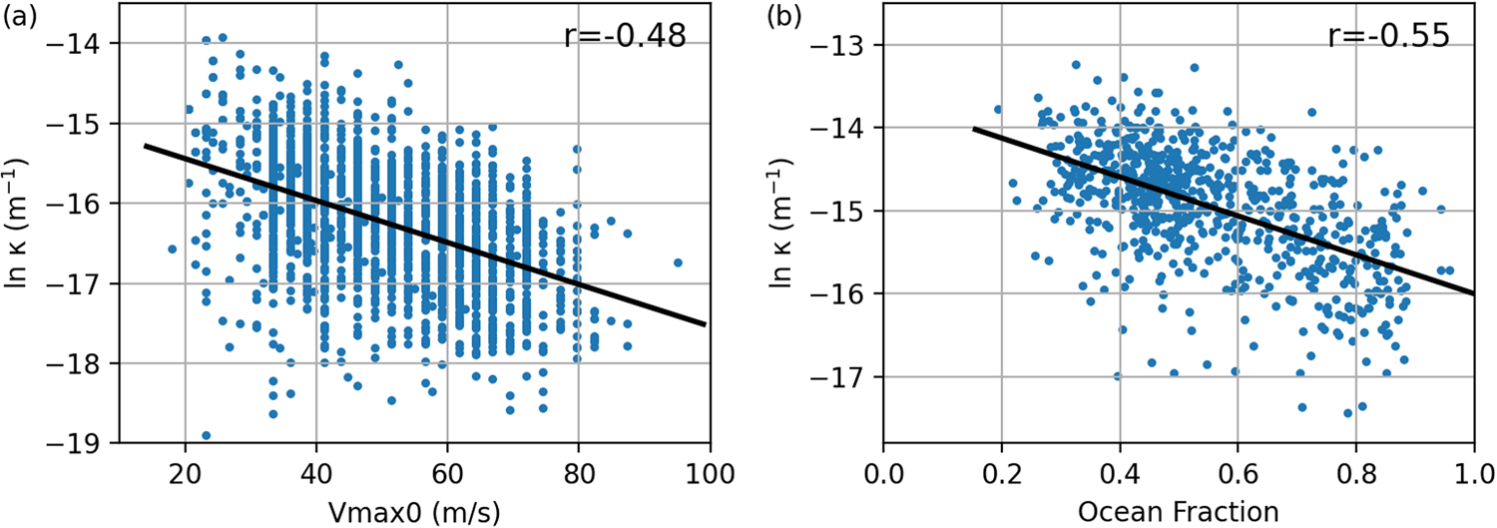
Decay parameter *k* against (**a**) initial wind speed for ocean decay, and (**b**) ocean fraction for land decay.

The constants *m*_*a*_, *c*_*a*_ and standard deviation, *σ*_*a*_, of the noise term, *ε*_*a*_, are determined through least squares fitting to the global observations. The data and fits are shown in Fig. 4a. The decay over land is treated differently. We find that the fractional surface area of ocean (i.e. not land), *F*_*O*_, in a three degree radius surrounding the point of landfall is strongly correlated with with the decay coefficient, with a larger landfall ocean fraction leading to a slower decay as expected^23^.

5 $$log\,\kappa ={m}_{b}{F}_{O}+{c}_{b}+{\varepsilon }_{b},{\varepsilon }_{b} \sim {\mathscr{N}}(0,{\sigma }_{b}^{2}).$$

where parameters are estimated via least squares regression as above and data and fits are shown in Fig. 4b.

#### Size

We use an axisymmetric modified Rankine wind speed profile^24^ given by,

6 $$V(r)=\left\{\begin{array}{cc}Vmax\left(\frac{r}{RMW}\right) & r\le RMW\\ Vmax{\left(\frac{RMW}{r}\right)}^{\alpha } & r > RMW\end{array}\right.$$

where *V*(*r*) is the wind speed at radius *r*, *Vmax* is the maximum wind speed, *RMW* is the radius of the maximum wind speed and *a* is a shape parameter and is defined by two measurements of wind speed at different radii (not within RMW). Since RMW and the radius of storm strength winds (~18 m/s), R18, are the most widely available size observations we choose these to give,

7 $$\alpha =\frac{log\frac{Vmax}{18}}{log\frac{R18}{RMW}}.$$

A weak dependence of TC size on intensity has been observed^25,26^, as has an important increase of size during the decay of a TC which contributes to the footprint of any damage^27^. We represent this behaviour within our model. For simplicity we calculate the TC size over the ocean as the TC wind speed decays from LMI to at least 25 m s^−1^. Below 25 m s^−1^ the shape parameter becomes ill-defined as Vmax approaches 18 m s^−1^ and R18 approaches RMW. The “final” size is near dissipation at Vmax = 25 m s^−1^. TCs have an initial size at LMI and final size at Vmax = 25 m s^−1^. The shape parameter is evaluated at LMI and Vmax = 25 m s^−1^ using Eq. 7. The results are not sensitive to the 25 m s^−1^ cut-off choice.

**Fig. 5:**
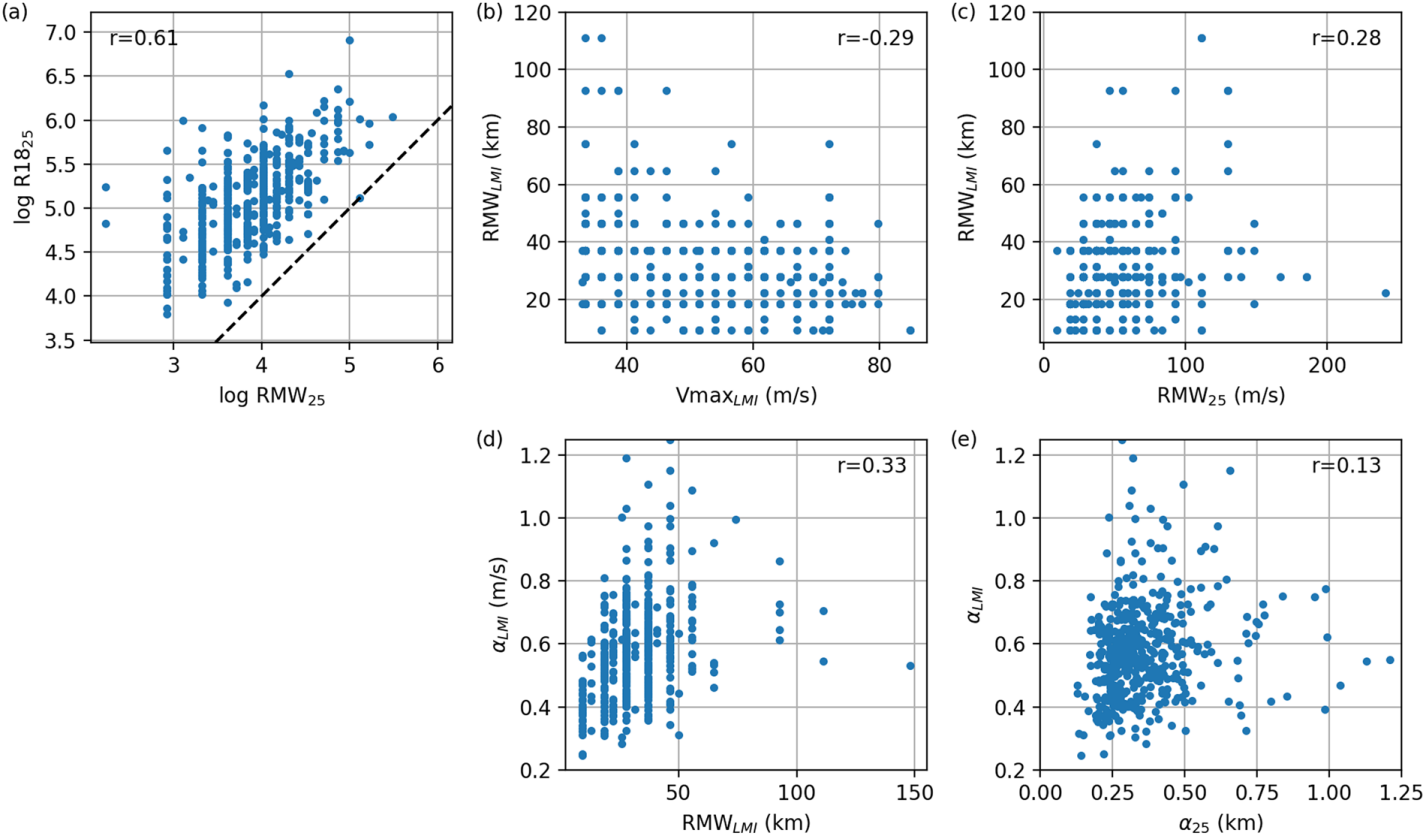
Dependence of (**a**) the radius of 18 m s^−1^ winds and RMW at Vmax = 25 m s^−1^; (**b**) radius of LMI and LMI; (**c**) RMW at LMI and at Vmax = 25 m s^−1^; (**d**) shape parameter *α*_*LMI*_ and radius of LMI; (**e**) shape parameter at LMI, *α*_*LMI*_, and shape parameter *α*_25_ at 25 m s^−1^.

We first characterise the final size parameters, *RMW*_25_ and *R*18_25_. They both have approximately log-normal distributions^28^ which can be justified theoretically^29^ and are significantly correlated (Fig. 5a). We therefore treat these quantities as joint log-normally distributed:

8 $$\left(\begin{array}{c}logRM{W}_{25}\\ logR1{8}_{25}\end{array}\right) \sim {\mathscr{N}}\,\left[\left(\begin{array}{c}{\mu }_{RM{W}_{25}}\\ {\mu }_{R1{8}_{25}}\end{array}\right),\left(\begin{array}{cc}{\sigma }_{RM{W}_{25}}^{2} & \rho {\sigma }_{RM{W}_{25}}{\sigma }_{R1{8}_{25}}\\ \rho {\sigma }_{RM{W}_{25}}{\sigma }_{R1{8}_{25}} & {\sigma }_{R1{8}_{25}}^{2}\end{array}\right)\right]$$

where *μ*, *σ* and *ρ* are the mean, standard deviation, and correlation of the logged quantities.

We find that radius of maximum wind at LMI, *RMW*_*LMI*_, is related to both Vmax at LMI and *RMW*_25_ (Fig. 5b,c):

9 $$RM{W}_{LMI}={m}_{1}LMI+{m}_{2}RM{W}_{25}+{c}_{1}+{\varepsilon }_{1},{\varepsilon }_{1} \sim {\mathscr{N}}(0,{\sigma }_{1}^{2}).$$

Similarly the initial shape parameter *α*_*LMI*_ is related to the initial RMW and *α*_25_ (Fig. 5d,e)):

10 $${\alpha }_{LMI}={m}_{3}RM{W}_{LMI}+{m}_{4}{\alpha }_{25}+{c}_{2}+{\varepsilon }_{2},{\varepsilon }_{2} \sim {\mathscr{N}}(0,{\sigma }_{2}^{2}),$$

where *m*_*i*_, *c*_*i*_ and *σ*_*i*_ are constants to be estimated through multiple linear regression to the observation data of the decay phase.

#### Wind pressure relationship

**Fig. 6:**
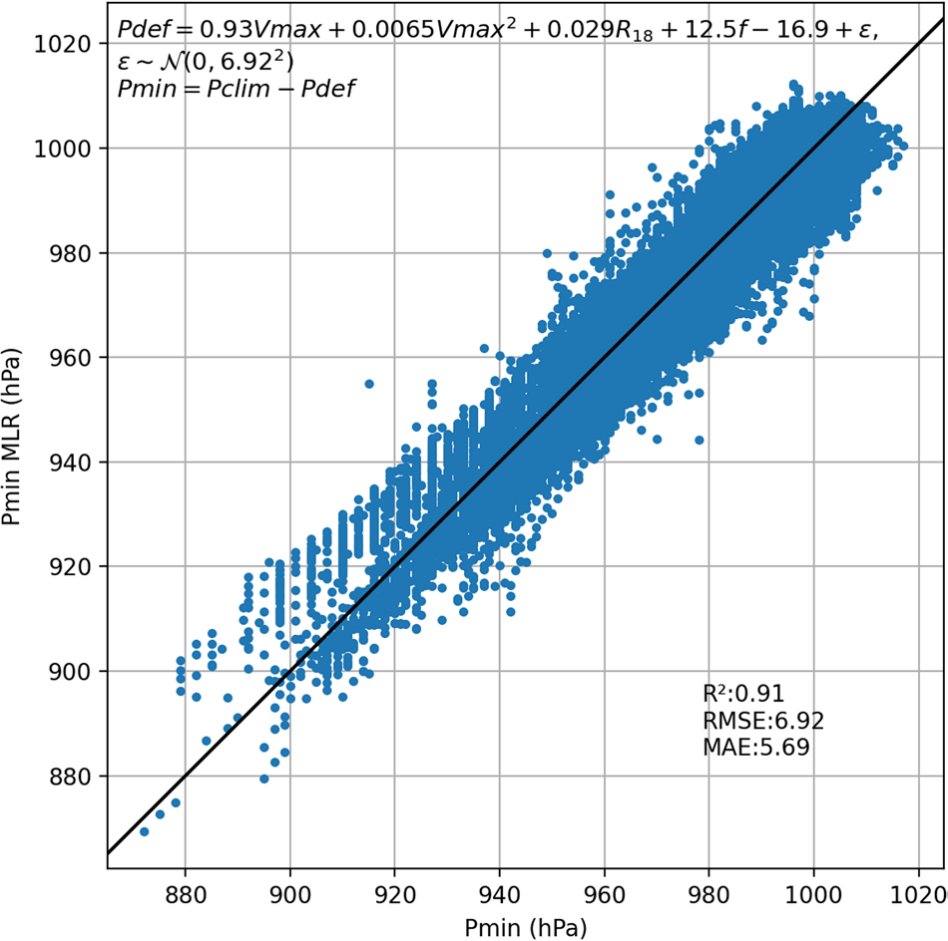
Minimum pressure calculated using Eq. 11 against observed minimum pressure for decay phase. Coefficients estimated with multiple linear regression.

Since IRIS is exclusively focused on post-LMI behaviour, we apply a new, unified relationship to determine the TC central minimum pressure across all basins for the decay phase. The size and latitude can modify the pressure wind relationship^30^ throughout the life-cycle, so we also include this effect:

11 $${P}_{def}=aVmax+bVma{x}^{2}+cR18+df+\varepsilon ,\varepsilon  \sim {\mathscr{N}}(0,{\sigma }^{2})$$

where *a,b,c,d* and *σ* are constants suitable for the decay estimated by multiple linear regression on the IRIS input data, *f* is the Coriolis parameter at the latitude of the TC, and *P*_*def*_ is the pressure deficit approximated by

12 $${P}_{def}={P}_{clim}-{P}_{min}$$

where *P*_*clim*_ is the monthly climatological pressure at the TC centre location. Figure 6 shows the observed and well-predicted central pressure during decay using the above equation.

### Simulation steps

Each basin and simulation year are generated independently. For a given year and basin, the number of TCs, *n*, is randomly sampled according to Eq. 1. Then *n* “parent” tracks are randomly chosen (with replacement) from that basin’s pool of historical input TCs (42 years’ worth). From each of these parent tracks (beginning at LMI), a stochastic “child” track is created by first perturbing the parent track location by constant longitude and latitude displacements drawn randomly from a normal distribution with zero mean and standard deviation of 1.0 degree. Then a displacement which grows in time at a constant rate drawn from a zero-mean normal distribution with standard deviation of 0.025 degrees per hour, reflecting the uncertainty cone growth, is applied as a subsequent perturbation. The child track is extended by extrapolating its translational motion based on its final 12 hours. Extrapolated motion relaxes to the monthly mean climatological steering wind (wind velocity at 500 hPa) on a timescale of five days. This can then represent the climatology of track curvature at higher latitudes, for example. The TC month is taken directly from the parent TC.

TC intensity simulation begins at LMI. First the climatological monthly mean PI at the month of the parent track and location of LMI is identified. The relative intensity (LMI/PI) is then sampled according to Eq. 2. Multiplying the relative intensity by PI then gives the LMI for a given TC. From LMI the intensity decays according to Eq. 3 with a decay constant given by Eq. 4. If the TC track makes landfall, a land decay constant is sampled via Eq. 5 and the TC decays accordingly. If the TC makes a subsequent seafall it decays with its original ocean decay constant. Some observed TCs have reintensified after making landfall and decaying, but this behaviour is not simulated in IRIS. If the TC moves to a location with PI below tropical storm intensity or to within 5 degrees of the equator the TC enters a fast exponential decay with a time constant of 6 hours. Decay continues until the TC intensity drops below tropical storm intensity.

Generating the size parameters is a three phase procedure. First the distribution specified in Eq. 8 is sampled from to produce a near-dissipation set of size parameters (*RMW*_25_, *R*18_25_) valid at Vmax = 25 m/s. This process may yield a non-physical combination (*RMW*_25_ > *R*18_25_), so to prevent this *R*18_25_ is set to be at least 1 km greater than *RMW*_25_. The size parameter, *α*, at Vmax = 25 m/s, is then calculated using Eq. 7. Then RMW and *α* at LMI are simulated using using Eqs. 9, 10 respectively, then *R*18_*LMI*_ is calculated via 7. This process may occasionally produce very large *R*18_*LMI*_ so these are capped at 600 km. Finally RMW and R18 are calculated for each time step along the track through linear interpolation. For values below Vmax = 25 m/s, R18 is calculated through 7 with *α* set to *α*_25_ to ensure R18 approaches RMW as Vmax approaches 18 m/s as required. The size influences the minimum pressure. Equations 11, 12 are used to generate *P*_*min*_ values with a single constant noise term per TC to maintain consistency.

The above method was used to generate 10,000 years of synthetic TCs in each of the six basins. The results of this simulation are analysed below.

## Data Records

The IRIS 10,000 year TC event set is available from figshare^31^. The data are stored in simple space-delimited text files with each line representing a single time step of a single TC. Each file has 1,000 years of TC data for a given basin. Descriptions of the columnar contents of the data are provided in Table 1.

**Table 1:** Description of columns in IRIS dataset.

#	Name	Unit	Description
1	ID		TC identifier, unique across entire dataset
2	Year		Starts at 0 every file
3	TC number		Starts at 0 every year
4	Month		
5	Time step		Starts at 0 every TC, interval 3 hr
6	Longitude	Deg	TC centre position
7	Latitude	Deg	TC centre position
8	Maximum wind speed	m/s	
9	Minimum pressure	hPa	
10	Radius of maximum winds	km	
11	Radius of gale force winds	km	

## Technical Validation

### Summary statistics

**Fig. 7:**
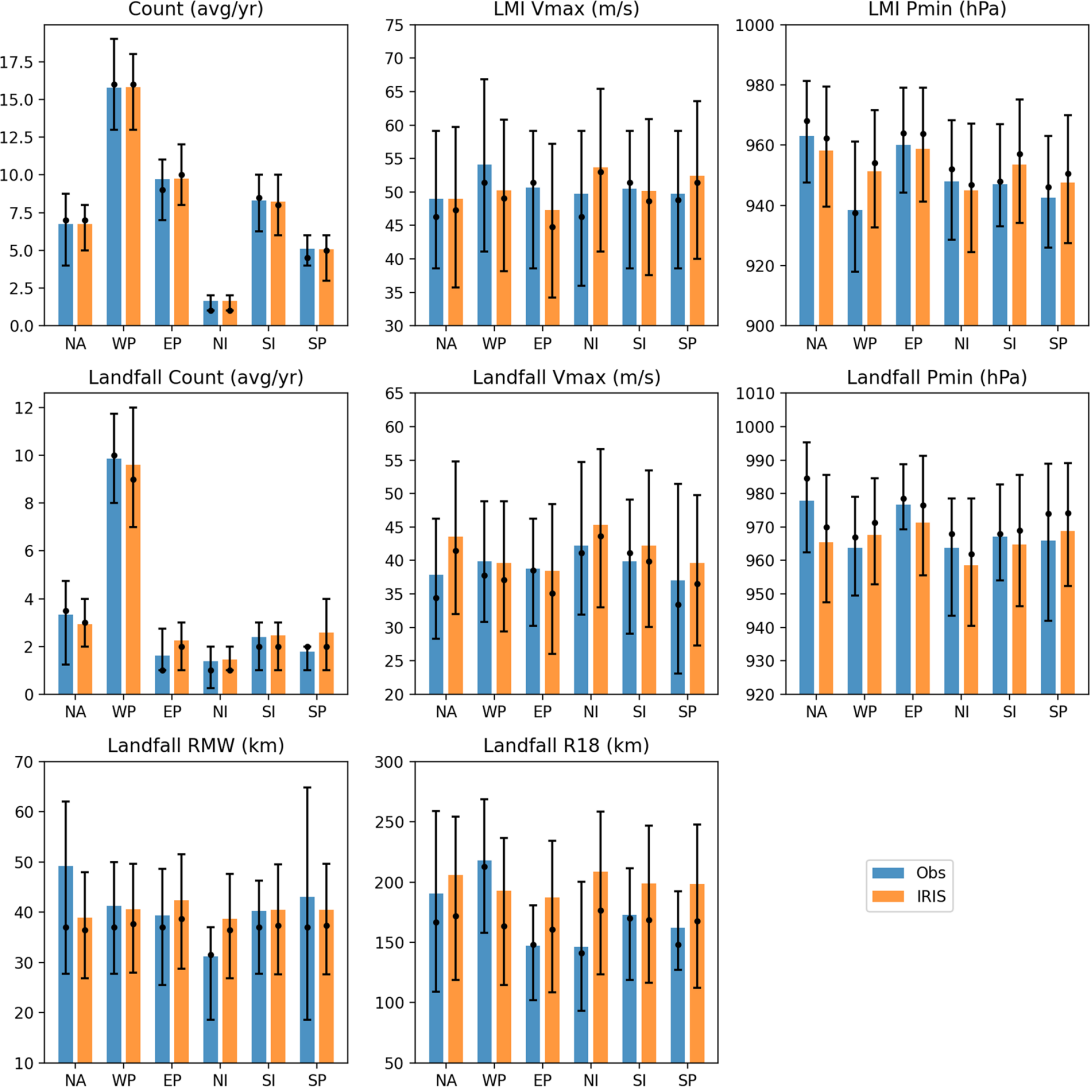
Comparison of observation (IBTrACS) and IRIS simulated summary statistics by basin. Shaded bars show mean values, black dot is the median and bars indicate the quartiles.

Since the IRIS model takes IBTrACS TC observation data as its input, we perform an initial validation by comparing summary statistics of the two. Key points in a TC lifecycle represented in IRIS are the LMI and landfall, if it occurs. We therefore present analysis of TC parameters at these points per basin and globally in Table 2 and Fig. 7 based on the 10,000 years of simulated IRIS data. Following a previous approach^10^, we do not intend to test for significant differences from the observations, but instead are satisfied if important TC risk metrics are broadly well-represented in IRIS.

**Table 2:** Summary statistic of observations (IBTrACS) and 10,000 years of IRIS simulation.

		NA	WP	EP	NI	SI	SP	GL
Count (avg/yr)	Obs	6.7 (4.0, 7.0, 8.8); 42	15.8 (13.0, 16.0, 19.0); 42	9.7 (7.0, 9.0, 11.0); 42	1.6 (1.0, 1.0, 2.0); 42	8.3 (6.2, 8.5, 10.0); 42	5.1 (4.0, 4.5, 6.0); 42	47.3 (42.0, 47.5, 51.0); 42
	IRIS	6.8 (5.0, 7.0, 8.0); 10000	15.8 (13.0, 16.0, 18.0); 10000	9.7 (8.0, 10.0, 12.0); 10000	1.6 (1.0, 1.0, 2.0); 10000	8.2 (6.0, 8.0, 10.0); 10000	5.1 (3.0, 5.0, 6.0); 10000	47.2 (42.0, 47.0, 52.0); 10000
LMI Vmax (m/s)	Obs	49.0 (38.5, 46.3, 59.1); 283	54.1 (41.1, 51.4, 66.8); 663	50.6 (38.5, 51.4, 59.1); 408	49.8 (36.0, 46.3, 59.1); 69	50.5 (38.5, 51.4, 59.1); 348	49.7 (38.5, 48.8, 59.1); 214	51.4 (38.5, 51.4, 61.7); 1985
	IRIS	49.0 (35.8, 47.3, 59.7); 67506	50.2 (38.2, 49.1, 60.8); 158047	47.3 (34.2, 44.8, 57.2); 97418	53.7 (41.1, 53.0, 65.4); 16400	50.1 (37.6, 48.6, 60.9); 82144	52.4 (40.0, 51.4, 63.6); 50647	49.8 (37.1, 48.2, 60.5); 472162
LMI Pmin (hPa)	Obs	963.0 (947.5, 968.0, 981.2); 280	938.4 (918.0, 937.5, 961.2); 312	960.1 (944.2, 964.0, 979.0); 298	948.0 (928.5, 952.0, 968.2); 40	947.1 (933.0, 948.0, 967.0); 165	942.6 (926.0, 946.0, 963.0); 90	951.5 (933.0, 955.0, 973.0); 1185
	IRIS	958.2 (939.6, 962.3, 979.5); 67506	951.3 (932.6, 954.0, 971.7); 158047	958.8 (941.2, 963.7, 979.0); 97418	944.9 (924.5, 946.8, 967.1); 16400	953.5 (934.2, 957.0, 975.2); 82144	947.6 (927.5, 950.5, 969.9); 50647	953.6 (934.6, 957.0, 974.9); 472162
Landfall Count (avg/yr)	Obs	3.3 (1.2, 3.5, 4.8); 42	9.9 (8.0, 10.0, 11.8); 42	1.6 (1.0, 1.0, 2.8); 42	1.4 (0.2, 1.0, 2.0); 42	2.4 (1.0, 2.0, 3.0); 42	1.8 (1.0, 2.0, 2.0); 42	20.4 (17.0, 19.5, 23.0); 42
	IRIS	2.9 (2.0, 3.0, 4.0); 10000	9.6 (7.0, 9.0, 12.0); 10000	2.2 (1.0, 2.0, 3.0); 10000	1.5 (1.0, 1.0, 2.0); 10000	2.5 (1.0, 2.0, 3.0); 10000	2.6 (1.0, 2.0, 4.0); 10000	21.3 (18.0, 21.0, 24.0); 10000
Landfall Vmax (m/s)	Obs	37.9 (28.3, 34.4, 46.3); 140	39.9 (30.8, 37.8, 48.8); 414	38.8 (30.2, 38.5, 46.3); 68	42.2 (31.9, 41.1, 54.7); 58	39.8 (29.0, 41.1, 49.1); 100	37.0 (23.1, 33.4, 51.4); 75	39.4 (29.3, 37.0, 48.8); 855
	IRIS	43.6 (32.0, 41.5, 54.8); 29313	39.6 (29.4, 37.1, 48.8); 96133	38.4 (26.0, 35.1, 48.4); 22473	45.3 (33.0, 43.6, 56.6); 14559	42.2 (30.0, 39.9, 53.5); 24551	39.6 (27.3, 36.5, 49.8); 25689	40.7 (29.5, 38.1, 50.8); 212718
Landfall Pmin (hPa)	Obs	977.8 (962.5, 984.5, 995.2); 140	963.7 (949.5, 967.0, 979.0); 207	976.6 (969.2, 978.5, 988.8); 54	963.8 (943.5, 968.0, 978.5); 31	967.2 (954.0, 968.0, 982.8); 50	965.9 (942.0, 974.0, 989.0); 33	969.4 (955.0, 974.0, 987.0); 515
	IRIS	965.4 (947.5, 969.9, 985.5); 29313	967.6 (952.8, 971.4, 984.6); 96133	971.3 (955.5, 976.5, 991.2); 22473	958.5 (940.4, 962.0, 978.6); 14559	964.7 (946.3, 969.0, 985.6); 24551	968.8 (952.4, 974.1, 989.1); 25689	966.9 (950.6, 971.2, 985.7); 212718
Landfall RMW (km)	Obs	49.2 (27.8, 37.0, 62.0); 75	41.2 (27.8, 37.0, 50.0); 187	39.4 (25.5, 37.0, 48.6); 32	31.2 (18.5, 31.5, 37.0); 29	40.2 (27.8, 37.0, 46.3); 45	43.1 (18.5, 37.0, 64.8); 31	41.9 (27.8, 37.0, 52.8); 399
	IRIS	39.0 (26.8, 36.5, 48.0); 29313	40.6 (28.0, 37.7, 49.6); 96133	42.4 (28.7, 38.7, 51.6); 22473	38.6 (26.8, 36.4, 47.6); 14559	40.4 (27.6, 37.4, 49.5); 24551	40.5 (27.6, 37.3, 49.7); 25689	40.4 (27.7, 37.5, 49.4); 212718
Landfall R18 (km)	Obs	190.4 (108.8, 166.7, 259.0); 63	218.1 (157.8, 213.0, 269.0); 196	147.1 (101.9, 148.2, 180.6); 31	146.1 (93.2, 141.2, 200.5); 30	172.8 (118.6, 169.9, 211.2); 46	162.1 (127.3, 148.2, 192.1); 29	193.2 (129.6, 180.6, 236.6); 395
	IRIS	205.8 (118.9, 172.0, 254.3); 29313	192.6 (114.6, 163.5, 236.5); 96133	187.2 (108.7, 160.7, 234.2); 22473	208.7 (123.5, 176.4, 258.6); 14559	198.9 (116.3, 168.5, 247.0); 24551	198.4 (112.4, 167.6, 247.9); 25689	196.4 (115.0, 166.2, 242.7); 212718

Mean and sample count given with 25th, 50th and 75th percentiles in brackets.

The IRIS mean annual counts per basin and globally match exactly those in IBTrACS because the Poisson count model takes a single parameter (mean count) from the input data, so over a long enough simulation, the counts will converge to those in the input data. The variability of annual count is also well-represented in IRIS indicating the Poisson assumption is adequate. The LMI behaviour is more complex and leads to small biases across the basins and a global bias of −1.6 m/s. This bias is likely due to the choice of distribution to represent the relative intensity (LMI/PI). The variability of LMI is also similar in both observations and simulation with a small bias of +0.2 m/s in the interquartile range globally. The minimum pressure, Pmin, at LMI also performs well in the simulation with a global bias of only +2.1 hPa and very similar interquartile range. There is a difference in the sign of the bias across basins, likely due to the choice of using a global pressure wind relationship, rather than using a per-basin formula as is common practice in best track type observation data.

Of greater importance from a risk perspective are characteristics of landfalling TCs. The annual frequency of landfalling TCs is well-represented, with a small positive bias of only +0.9 landfalls per year globally in IRIS compared to the observations. This bias compares favourably with others^10^. The mean maximum wind speed at landfall is 39.4 m/s in the observations, compared to 40.7 m/s in IRIS. The sign of the bias varies across basins. The interquartile range of Vmax at landfall is similar in IRIS (21.3 m/s) to the observations (19.5 m/s). Pmin at landfall has a small global bias of −2.5 hPa but again the sign differs across basins as above.

The global mean radius of maximum wind speed, RMW, is slightly smaller modelled (40.4 km) than observed (41.9 km). In contrast the model global mean radius storm-strength winds, R18, at landfall is a bit larger (196 km) compared to the observations (193 km). The sign of the bias differs across basins. The discrepancy is likely the result of several factors. It is worth noting that the size parameterisation is global, so differences in size between basins is not explicitly accounted for. Furthermore, the size parameterisation is not fitted to landfall data but simulated. Finally any bias in mean landfall Vmax will also affect the landfall size.

### Spatial coverage

**Fig. 8:**
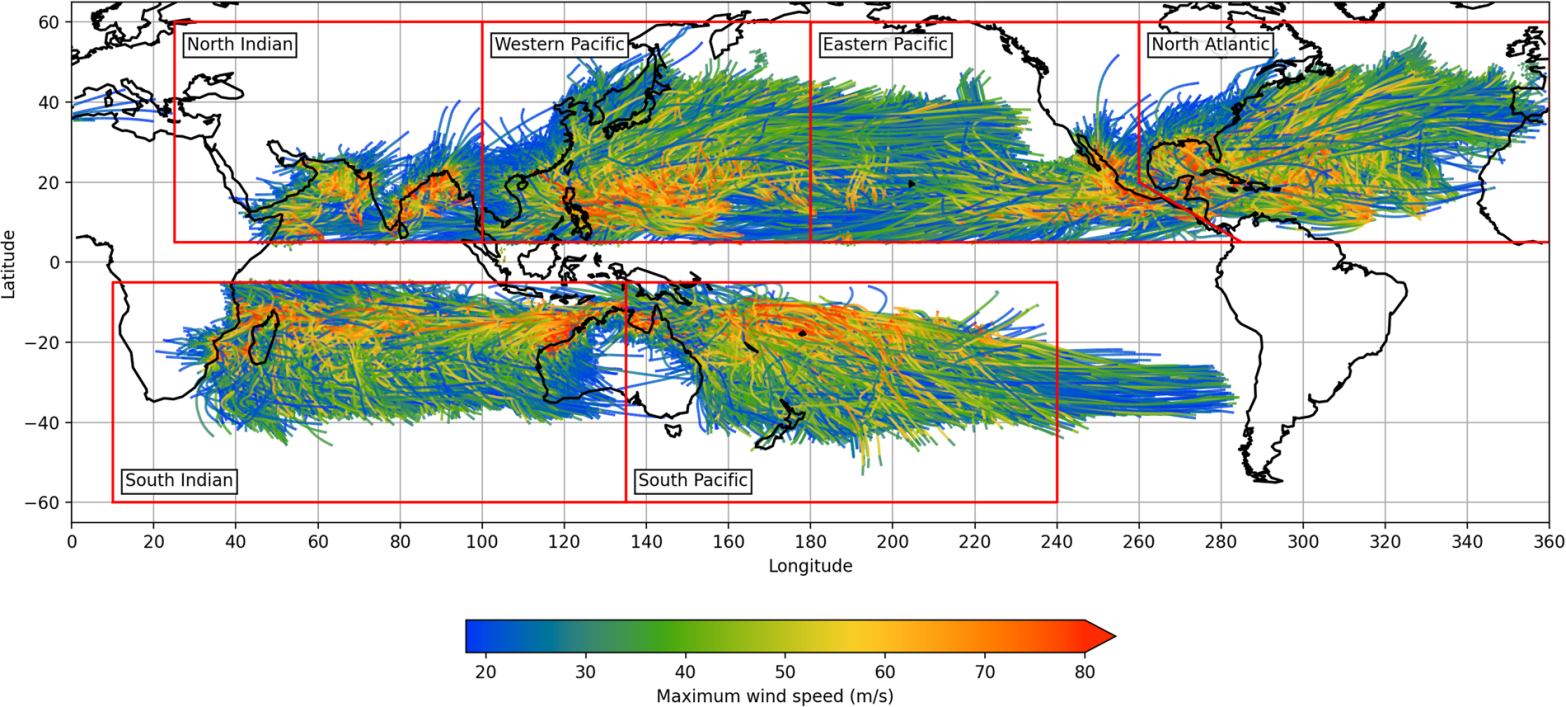
1000 years of IRIS simulated tracks and maximum wind speed.

A primary purpose of synthetic TC track data is to fill in the gaps in observed TC tracks which exist due to the limited period or reliable historical observation data. Figure 8 shows the tracks of a sample of 1000 years of IRIS output and maybe compared with the 42 years of observed tracks in Fig. 1. The IRIS tracks form a much more comprehensive coverage of the major TC regions with all vulnerable coastal areas densely populated with tracks allowing for a good analysis of risk not possible in the observation set. IRIS has very few very long tracks extending beyond their origin basin. Some tracks extend inland over continents further than in the observed tracks. This is a natural consequence of the statistical extrapolation performed by this type of model where not all relevant physics is present. For example, ocean decays are terminated when the MPI drops below tropical storm intensity, typically at higher latitudes, but this measure does not include, for example, a vertical wind shear term which may result in earlier and faster dissipation. For decay over land, no explicit adjustment is made for orographic effects which again may lead to earlier dissipation and reduce continental penetration. Overall IRIS performs as desired, increasing the coverage and density of tracks while preserving the basic observed pattern.

### Return period analysis

**Fig. 9:**
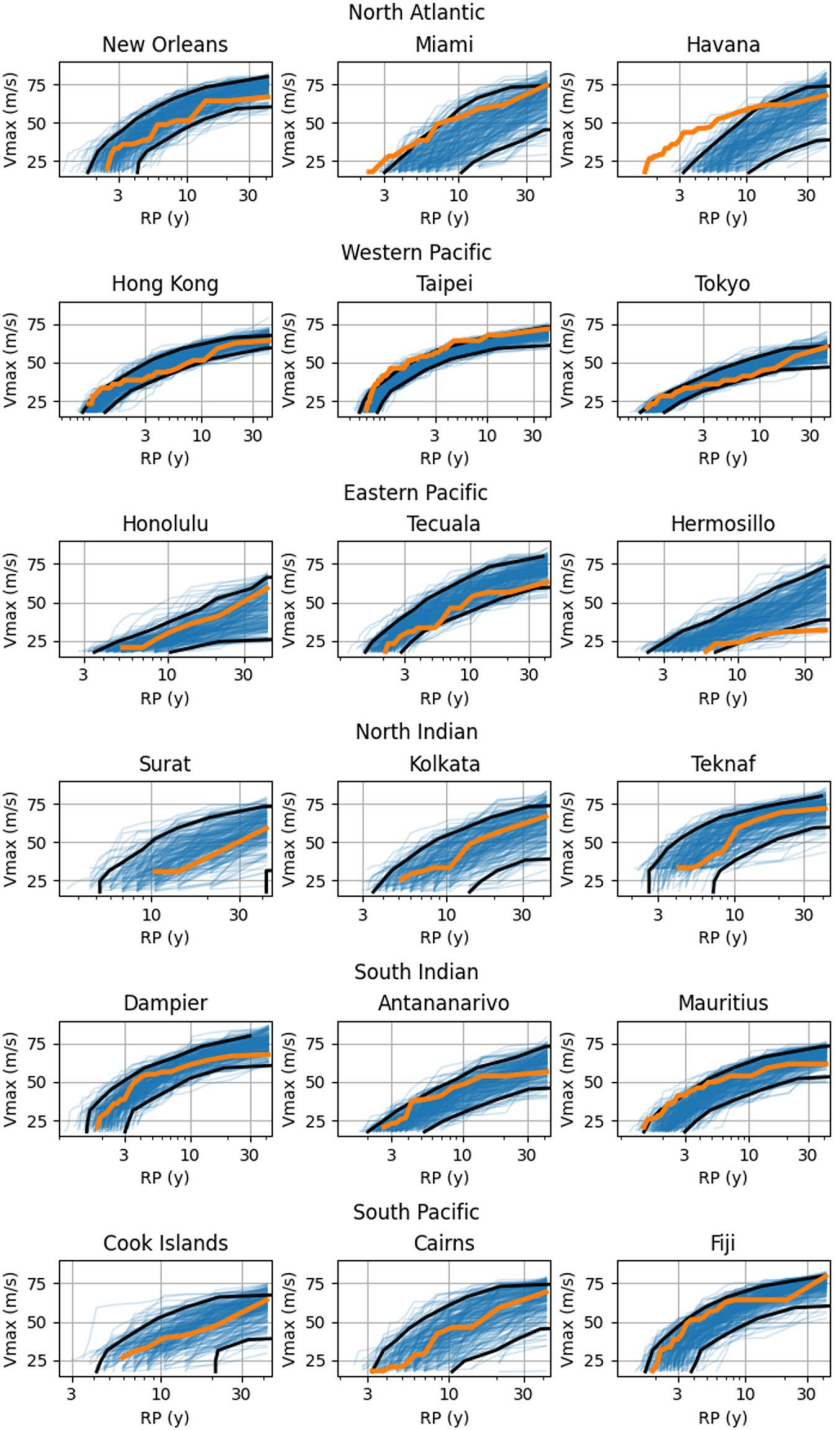
Maximum wind speed as a function Return Period (RP) of TCs within 2 degree radius of location for 238 ensembles of 42 years (9,996 years in total) of IRIS in (blue) and 42 years of IBTrACS observations (orange) at three sample locations per basin. Black lines show the 5th and 95th percentiles of return period across IRIS 42-year ensembles.

Perhaps the most important validation concept from a risk perspective is that of the return period and return value. The return period is the inverse occurrence frequency of an event of at least a given magnitude, the return value. The shape of return period curves are sensitive to the upper tails of the return value distribution. By comparing IRIS and observed return period we can asses the ability of IRIS to produce extreme events statistically compatible with those observed. We split the 10,000 years of synthetic tracks into 238 ensembles of 42 years, which is the time span of observations. The return curves for events within 2° of three sample locations vulnerable to landfalling TCs in each basin were calculated. The spread of these curves then offers an estimate of the sampling error present in the observed curves. Observed and simulation ensemble curves are shown Fig. 9. First, it is clear from this analysis that IRIS is capable of generating events outside the range of the input data. Observed return values are generally within the range of the simulated ensemble return values across all return periods in all basins. In some locations (e.g. Honolulu, Fiji) the observed return values are toward the upper range of the simulated values. In others (e.g. New Orleans, Hong Kong) observations are towards the lower end of the simulations. This may be due to model bias in these areas or due to local sampling uncertainty in the observation record bearing in mind that the observations are only one outcome of plausible histories.

Given that IRIS compares well with the observations in this extreme event validation which is more relevant than the mean summary statistics, we can state that the IRIS dataset is suitable for the purposes of TC hazard and risk analysis.

## Usage Notes

The IRIS dataset^31^ is based on the mean of the 42-year input observations. The 10,000 year output is fixed to the “current” 1980–2021 TC climate rather than a prediction of any sort. The IRIS model produces independent synthetic years, so the dataset has no inter-annual correlation which may be important for some applications, for example, calculating multi-year risk. The magnitude of the inter-annual variability in the dataset resembles that of the 42-year observation period which may not contain the full range of the multidecadal variability of the Earth climate system.

Furthermore, the observations upon which the IRIS dataset is based represent only one realisation of the 1980–2021 climate and as such is a limited sample. We have tested the sensitivity of IRIS to removing significant observed events from its input set (not shown) and find the results are robust on regional and sub-regional scales. However we do still expect some impact of the limited observation sample size on the IRIS dataset and note that it is representative only of the observed climatology.

Basins are simulated independently so any inter-basin dependence which may be present in the observations are not present in IRIS. This may lead to over- or underestimates of TC metrics when basins are aggregated.

Some studies suggest there may have been a poleward shift of LMI in some basins. We do not attempt to model trends in LMI location, or any TC phenomena, over the observation period and hence the output may not be representative of the current climate in 2023, but rather the mean climate of the observation period.

Care should be taken interpreting the dataset in regions where it is likely the storm systems may no longer be considered tropical, i.e. at high latitudes. The dataset is not intended to represent non-tropical systems, and since extra- and post-tropical systems were excluded from the fitting process and input track data, the decay and size models may not be appropriate for these storms. There is no treatment of extratropical transition in the model.

Finally, the IRIS track model is based on perturbing observed tracks by an amount consistent with the error in current TC track forecasts. The forecast error of TC tracks has reduced over time as models have improved but one study suggests that we may have reached the limit of predictability of TC tracks^32^. Therefore, although somewhat subjective, the current forecast error cone may be considered an estimate of the inherent unpredictability of TC tracks, but we accept this may change over time. Furthermore, the data we used to define the cone extend only to 5 days and the assumption of linear growth may not be true beyond this. However, we note that all the validation metrics presented above are insensitive to this choice of track perturbation model and applying no error-cone perturbation to the “parent” track changes simulated global mean landfall rates and intensities by less than 1% and basin values by less than 2%. However, some regions when examined on smaller spatial scales may be more sensitive to the choice of track perturbation model.

## Data Availability

The IRIS code is publicly available (https://github.com/njsparks/iris) and a release of the version described here has been archived^33^.
